# Adoecimento mental de ex-moradores afetados pela extração de sal-gema de uma mineradora de Maceió, Alagoas, Brasil

**DOI:** 10.1590/0102-311XPT209523

**Published:** 2025-01-13

**Authors:** Priscilla Souza dos Santos, Verônica de Medeiros Alves, Ana Paula Nogueira de Magalhães, Adriana Inocenti Miasso, Matheus William de Oliveira Melo

**Affiliations:** 1 Universidade Federal de Alagoas, Maceió, Brasil.; 2 Escola de Enfermagem de Ribeirão Preto, Universidade de São Paulo, Ribeirão Preto, Brasil.

**Keywords:** Transtornos Mentais, Ideação Suicida, Desastres Provocados pelo Homem, Assistência à Saúde, Mental Disorders, Suicidal Ideation, Man-Made Disasters, Health Care, Trastornos Mentales, Ideación Suicida, Desastres Provocados por el Hombre, Atención a la Salud

## Abstract

Este estudo tem como objetivo investigar indícios de adoecimento mental em vítimas da instabilidade do solo nos bairros afetados pela extração de sal-gema de uma mineradora localizada na cidade de Maceió, Alagoas, Brasil. É um estudo quantitativo, descritivo-analítico e transversal. A amostra foi intencional e não probabilística e consistiu em 158 participantes, com “poder de efeito” de 0,50 (efeito de tamanho moderado) e nível de confiança de 99,9%. Foi disponibilizado um *link* em redes sociais com convite para participar da pesquisa online. A maioria dos participantes era adulta, do sexo feminino, preta/parda, elevado nível de escolaridade e relatou piora percebida na renda mensal, na saúde física e mental e na forma como veem sua vida após a realocação de suas residências. A maioria dos participantes apresentou sintomas depressivos (87,34%), ansiedade moderada e grave (55,7%) e rastreio positivo para transtornos mentais comuns (77,22%). Ao analisar a percepção atual das mudanças que aconteceram na vida dos ex-moradores antes e após serem realocados devido à instabilidade do solo, identificou-se que, após a realocação, aqueles com maior demanda psiquiátrica e ideação suicida apresentaram prevalência mais elevada para sintomas depressivos, sintomas de ansiedade grave e rastreio positivo para transtornos mentais comuns. Esta pesquisa destaca a importância de se pensar em políticas, estratégias e ações de prevenção dos impactos na saúde mental, na recuperação e na reabilitação da saúde em cenários de desastres socioambientais. Implementar medidas preventivas e oferecer suporte psicológico e social adequado pode reduzir os efeitos prejudiciais desses desastres sobre as pessoas afetadas.

## Abstract (en)

This study aimed to investigate the presence of mental illness in victims of soil instability in neighborhoods affected by rock salt extraction from a mining company located in the city of Maceió, Alagoas, Brazil. It is a quantitative, descriptive-analytical, and cross-sectional study. The sample was intentional and non-probabilistic and consisted of 158 participants, with a 0.50 “power of effect” (moderate size effect) and a 99.9% confidence level. A link was made available on social media with an invitation to participate in the online survey. Most participants were adult, female, black/mixed-race, with a high level of education, and reported a perceived worsening in monthly income, physical and mental health, and in the self-perception of their lives after the relocation of their homes. Most participants had depressive symptoms (87.34%), moderate and severe anxiety (55.7%), and positive screening for common mental disorders (77.22%). By analyzing the current perception of the changes in the lives of former residents before and after being relocated due to the ground instability, it was identified that, after relocation, those with greater psychiatric demand and suicidal ideation had a higher prevalence of depressive symptoms, symptoms of severe anxiety, and positive screening for common mental disorders. This research highlights the importance of elaborating policies, strategies and actions to prevent impacts on mental health, recovery, and health rehabilitation in scenarios of socio-environmental disasters. Implementing preventive measures and offering adequate psychological and social support can reduce the detrimental effects of such disasters on the affected population.

## Abstract (es)

El estudio tiene como objetivo investigar la presencia de enfermedades mentales en víctimas de la inestabilidad del suelo en barrios afectados por la extracción de sal de roca de una empresa minera ubicada en la ciudad de Maceió, Alagoas, Brasil. Se trata de un estudio cuantitativo, descriptivo-analítico y transversal. La muestra fue intencional y no probabilística y estuvo compuesta por 158 participantes, con un “poder del efecto” de 0,50 (efecto de tamaño moderado) y un nivel de confianza del 99,9%. Se facilitó un enlace en las redes sociales con una invitación a participar en la encuesta en línea. La mayoría de los participantes era adulto, mujer, negra/parda, con un alto nivel de educación y reportaron percibir un empeoramiento en sus ingresos mensuales, en su salud física y mental y en la forma en que ven sus vidas después de reubicar sus hogares. La mayoría de los participantes presentó síntomas depresivos (87,34%), ansiedad moderada y severa (55,7%) y detección positiva de trastornos mentales comunes (77,22%). Al analizar la percepción actual de los cambios que se produjeron en la vida de los exresidentes antes y después de ser reubicados debido a la inestabilidad del suelo, se identificó que tras la reubicación, aquellos con mayores demandas psiquiátricas e ideación suicida tuvieron mayor prevalencia de síntomas depresivos, síntomas de ansiedad severa y detección positiva de trastornos mentales comunes. Esta investigación resalta la importancia de pensar políticas, estrategias y acciones para prevenir impactos en la salud mental, recuperación y rehabilitación de la salud en escenarios de desastres socioambientales. Implementar medidas preventivas y ofrecer apoyo psicológico y social adecuado puede reducir los efectos nocivos de estos desastres en las personas afectadas.

## Introdução

No dia 3 de março de 2018, um abalo sísmico de magnitude 2,4 foi detectado pelas estações da Rede Sismográfica Brasileira na cidade de Maceió, Estado de Alagoas, Brasil. O tremor foi notado pelos moradores dos bairros Serraria, Pinheiro, Cruz das Almas, Farol e Jatiúca ^1^. Posteriormente, identificou-se o surgimento de rachaduras no solo, em edifícios e casas de alguns bairros, havendo a necessidade de investigação desse fenômeno pelo Serviço Geológico do Brasil (SGB). A realização de diversos estudos e análises revelou a ocorrência de desestabilização das cavidades provenientes da extração de sal-gema, com consequente afundamento do terreno e trincas no solo e nas edificações em parte dos bairros Pinheiro, Mutange e Bebedouro ^2^.

A atividade de exploração de sal-gema é realizada pela Braskem, a sexta maior petroquímica do mundo, com 41 unidades industriais em quatro países (Brasil, Estados Unidos, México e Alemanha) e capacidade anual de produção de 9,3 milhões de toneladas de resinas termoplásticas (polietileno, polipropileno e policloreto de vinila) e 10,7 milhões de toneladas de químicos básicos (como eteno, propeno, butadieno, benzeno, entre outros) ^3^.

Em maio de 2019, a Braskem interrompeu a extração de sal-gema em Maceió e paralisou a fábrica de cloro-soda, no Pontal da Barra. Foi criado o Programa de Compensação Financeira e Apoio à Realocação (acordo entre Braskem, Ministério Público Federal, Ministério Público do Estado de Alagoas, Defensoria Pública da União e Defensoria Pública do Estado de Alagoas). O acordo prevê medidas para mitigar, reparar e compensar os impactos do fenômeno geológico nos bairros afetados: Pinheiro, Mutange, Bebedouro, Bom Parto e Farol ^4^.

Mais de 60 mil pessoas estão em processo de migração forçada, em decorrência da extração irresponsável de sal-gema em solo urbano ^5^, deixando para trás suas residências, construídas durante décadas e que eram espaços de convivência familiar, com vizinhos e amigos. A indenização recebida pelos ex-moradores tem sido considerada como venda compulsória do terreno para a Braskem, que terá sua posse. Estudo realizado com essa população mostra que alguns aspectos necessitam de maior atenção, como a ausência de um sistema de acompanhamento das etapas da negociação, ausência de equipe de avaliação do imóvel, proposta de indenização baseada na avaliação individual do imóvel e apoio às famílias de acordo com o padrão de vida estabelecido ^4^.

Os desastres ambientais se relacionam aos socioambientais, por afetarem diretamente o meio ambiente e todo o seu entorno populacional. Tem-se como exemplo o caso do rompimento de barragens de rejeito de minérios, no desastre de Brumadinho, Minas Gerais. Esses desastres são irreparáveis e irreversíveis, resultando em consequências relevantes para o meio ambiente, para a saúde física da população e, em longo prazo, para sua saúde mental ^6^.

Estudo sobre a desterritorialização e o sofrimento social da população, decorrente do rompimento da barragem de mineração no Município de Mariana, em Minas Gerais, identificou a marcante relação simbólica da população com o seu lugar de origem, traduzida na forma de vínculos e pertencimento ^7^.

Além do medo e da insegurança, as pessoas que perderam seus lares devido a desastres experimentam tristeza e angústia ^8^. Os indícios de adoecimento mental foram identificados em diversos estudos realizados com populações vítimas de desastres naturais ^9^ ^,^ ^10^ ^,^ ^11^. Todavia, não há dados disponibilizados por órgãos de saúde pública ou pela Braskem sobre o impacto da instabilidade do solo ocorrida na cidade de Maceió na saúde mental das pessoas realocadas, dificultando sua mensuração ^12^.

Considerando as evidências científicas do impacto de desastres socioambientais na saúde mental e a experiência vivenciada pela população de Maceió diante da necessidade de mudança de suas residências, tem-se como questão de pesquisa: há adoecimento mental em pessoas vítimas da instabilidade do solo nos bairros afetados pela extração de sal-gema de uma mineradora localizada na cidade de Maceió?

Destaca-se que não existem estudos relacionados a essa questão, evidenciando a relevância e ineditismo desta pesquisa. Nesse contexto, o objetivo deste trabalho foi investigar o adoecimento mental de vítimas desse desastre.

## Métodos

Trata-se de estudo quantitativo, descritivo-analítico e transversal, norteado pelo *Checklist* de *Verificação para Relatar Resultados de Pesquisas Eletrônicas na Internet* (*The Checklist for Reporting Results of Internet E-Surveys −* CHERRIES). É um estudo do tipo *survey*, que visa coletar dados sobre uma determinada amostra de indivíduos por meio de questionários estruturados.

Este estudo faz parte do projeto *Adoecimento Mental das Pessoas Vítimas da Instabilidade do Solo nos Bairros do Pinheiro, Mutange e Bebedouro em Maceió, Alagoas*. Foi aprovado pelo Comitê de Ética em Pesquisa da Universidade Federal de Alagoas (parecer nº 5.543.398 e CAAE: 58114022.0.0000.5013), em 25 de julho de 2022.

Segundo o programa de compensação financeira e apoio à realocação (criado após acordo entre a Braskem, Ministério Público Federal, Ministério Público do Estado de Alagoas, Defensoria Pública da União e Defensoria Pública do Estado de Alagoas), estima-se que até setembro de 2021, 35 mil pessoas foram realocadas dos bairros afetados para outras regiões ^13^.

A amostra foi intencional e não probabilística. Os critérios de inclusão para a participação neste estudo foram: ter idade ≥ 18 anos e ter residido em bairros afetados e em residências desocupadas devido à instabilidade do solo. Foram considerados como critérios de exclusão: ter saído de sua residência em bairros afetados por outro motivo que não seja a desocupação por instabilidade do solo.

Inicialmente, foi realizado o cálculo amostral no programa Epi Info versão 7.2.4 (https://www.cdc.gov/epiinfo/index.html), considerando uma população de 35 mil pessoas. Foi utilizado 5% de margem de erro e intervalo de 95% de confiança (IC95%). Por ser um estudo inédito, a frequência esperada foi de 50%. Com isso, o valor da amostra resultou em 380 participantes. Como a adesão ao estudo foi baixa, realizou-se uma análise de poder com a amostra obtida. A análise de poder é uma técnica estatística utilizada para determinar a capacidade de um estudo de detectar um efeito estatisticamente significativo, caso ele exista. Essa análise foi conduzida com o auxílio do software G*Power versão 3.0 (http://www.psycho.uni-duesseldorf.de/abteilungen/aap/gpower3), por meio de uma análise *post-hoc*, realizada após a coleta dos dados. O objetivo era verificar se o estudo tinha participantes suficientes para identificar diferenças estatisticamente significativas ^14^.

Neste estudo, a análise de poder *post-hoc* revelou um poder de 99,9% para uma amostra de 158 participantes, com um tamanho de efeito de 0,5. Isso indica uma alta probabilidade de que o estudo seja capaz de detectar diferenças estatisticamente significativas, se existirem, com base nessa amostra. O tamanho de efeito de 0,5 refere-se à magnitude do efeito que o estudo pode detectar, sendo considerado um efeito moderado.

Para coleta dos dados, um link da pesquisa no software *online* QuestionPro (https://www.questionpro.com/pt-br/) foi disponibilizado na rede social Instagram. Foi criado um perfil, denominado @vidas_rachadas, para facilitar a divulgação do estudo. Foram realizadas divulgações da pesquisa, do perfil do Instagram e do *link* de acesso por meio de reportagens na TV e em jornais impressos e *online* ^15^ ^,^ ^16^ ^,^ ^17^ ^,^ ^18^ ^,^ ^19^.

Ao selecionarem o *link* de acesso à pesquisa, imediatamente os interessados acessaram o Termo de Consentimento Livre e Esclarecido (TCLE), com informações sobre o objetivo, a metodologia, as contribuições do estudo e um endereço de e-mail que permitia a comunicação para tirar dúvidas e receber orientações das pesquisadoras. Após aceitarem participar do estudo, o software direcionava para o preenchimento dos instrumentos de coleta de dados na mesma plataforma.

Após conclusão das respostas, foi solicitado aos entrevistados que divulgassem com outros ex-moradores a pesquisa. Essa técnica de amostragem não probabilística denominada *snowball*, ou bola de neve, auxilia na realização da coleta de dados em populações com dificuldade de acesso, de forma que a amostra cresce conforme os entrevistados indicam outras pessoas para participarem da pesquisa ^20^.

Foram utilizados os seguintes instrumentos: (1) questionário de identificação e dados sociodemográficos; (2) Escala de Depressão (CES-D, do original: *The Center for Epidemiological Studies-Depression Scale*), (3) *Inventário de Ansiedade de Beck*; e (4) *Questionário de Autorelato* (*Self Report Questionnaire,* SRQ-20).

O questionário de identificação e dados sociodemográficos foi utilizado para conhecer as particularidades da amostra e o contexto. O CES-D é composto por 20 itens que questionam sintomas depressivos nos sete dias anteriores ao preenchimento do instrumento. Cada resposta admite quatro gradações crescentes de intensidade: nunca ou raramente; às vezes; frequentemente; e sempre, sendo atribuídas as pontuações 0, 1, 2 e 3, respectivamente. As respostas de todos os itens são somadas, sendo a pontuação total de 15 pontos o nível de corte que indica a presença de sintomas depressivos significativos ^21^.

O *Inventário de Ansiedade de Beck* consiste numa escala de autorrelato que mensura a intensidade de sintomas de ansiedade. O inventário contém 21 itens que devem ser avaliados pelo sujeito com referência a si mesmo, em uma escala de quatro pontos. A pessoa deve considerar como tem se sentido na última semana, incluindo o dia de sua aplicação, com opções de 1 a 4 em que cada número representa as classificações: 1 - absolutamente não; 2 - levemente: não me incomodou muito; 3 - moderadamente: foi muito desagradável, mas pude suportar; e 4 - gravemente: dificilmente pude suportar. O escore total é obtido a partir da soma dos escores dos itens individuais, que permite a classificação em níveis de intensidade da ansiedade mínima, leve, moderada ou grave ^22^.

O SRQ-20 foi elaborado por Harding et al. ^23^ e validado no Brasil por Mari & Willians ^24^. É composto por 20 itens elaborados para a identificação de transtornos mentais comuns (TMC). Os escores alcançados indicam a probabilidade de existência de TMC ou de desconforto emocional, a partir do intervalo 0 (nenhuma probabilidade) a 20 (extrema probabilidade). Para esta pesquisa, o ponto de corte adotado foi baseado no estudo de Mari & Willians ^24^, no qual sete ou mais respostas positivas indicam presença de TMC.

Inicialmente, os dados foram descritos por meio de frequências absolutas e percentuais (variáveis qualitativas) e de medidas como média, desvio padrão (DP), mínimo, mediana e máximo (variáveis quantitativas). Para verificar o efeito da intervenção em relação às variáveis qualitativas, foi proposto o teste de McNemar ^25^.

Foram realizadas perguntas para analisar a percepção de mudanças que aconteceram na vida das pessoas antes e após serem realocadas devido à instabilidade do solo. A avaliação foi realizada por meio da repetição da mesma pergunta, permitindo ao participante avaliar, mediante sua percepção, como se apresentava determinada situação em sua vida. As variáveis avaliadas antes e após a realocação devido à instabilidade do solo foram: renda mensal da família; número de pessoas que moram na casa; como considera sua saúde física e mental; faz acompanhamento psicológico e psiquiátrico; tem pensamentos de tirar sua vida; e como considera sua vida. Nesse contexto, para estimar a razão de prevalência (RP) bruta, foi utilizado o modelo de regressão de Poisson com variância robusta ^26^.

Todas as análises foram realizadas com o auxílio do software SAS 9.4 (https://www.sas.com/). Para todas as análises, adotou-se um nível de 5% de significância.

## Resultados

O *link* do questionário desta pesquisa foi visualizado por 1.876 pessoas, mas apenas 383 o responderam. No entanto, houve 212 desistências durante o preenchimento e 13 recusas em participar do estudo. Dessa forma, obteve-se um total de 158 participantes, com uma taxa de conclusão das respostas de 44,65%. O tempo médio para o preenchimento do questionário foi de oito minutos.

A média de idade dos participantes foi de 42,06 (±12,97) anos. A maioria era do sexo feminino (74,68%), parda/preta (61,78%) e branca (35,67%), com Ensino Superior incompleto, completo e pós-graduação (84,95%), servidor público (27,74%) ou desempregado (17,42%). Quanto à distância entre a atual residência e o trabalho, a maioria relatou que era muito distante (46,46%) ou um pouco mais distante (30,71%) do que quando morava no bairro afetado pelo afundamento do solo. A maioria dos entrevistados residia no bairro Pinheiro (65,38%) (Tabela 1).

**Tabela 1:** Características sociodemográficas e aspectos da saúde mental das pessoas vítimas da instabilidade do solo nos bairros afetados pela extração de sal-gema de uma mineradora de Maceió, Alagoas, Brasil, 2023.

Variáveis	n	%
Sexo		
Feminino	118	74,68
Masculino	40	25,32
Total	158	100,00
Identificação em relação a sua cor de pele		
Branca	56	35,67
Preta	22	14,01
Parda	75	47,77
Amarela	3	1,91
Indígena	1	0,64
Total	157	100,00
Nível de escolaridade		
Nunca estudou/Analfabeto	1	0,88
Ensino Fundamental incompleto	4	3,54
Ensino Fundamental completo	1	0,88
Ensino Médio incompleto	8	7,10
Ensino Médio completo	2	1,77
Ensino Superior incompleto	31	27,43
Ensino Superior completo	28	24,78
Pós-graduação	37	32,74
Outros	1	0,88
Total	113	100,00
Situação de trabalho atual		
Empregado(a) com carteira assinada	26	16,77
Empregado(a) sem carteira assinada	13	8,39
Trabalha por conta própria e não tem empregados	16	10,32
Empregador(a)	5	3,23
Servidor(a) público	43	27,74
Aposentado(a)	9	5,81
Pensionista	2	1,29
Do lar	6	3,87
Desempregado	27	17,42
Outro	8	5,16
Total	155	100,00
Como você considera o trajeto de sua residência atual para o seu local de trabalho?		
Muito distante	59	46,46
Um pouco mais distante	39	30,70
Não houve mudança	15	11,81
Mais próximo que o trajeto que realizava na minha residência anterior	9	7,09
Muito próximo	5	3,94
Total	127	100,00
Em que bairro você residia?		
Pinheiro	102	65,38
Bebedouro	35	22,44
Mutange	8	5,13
Outro	11	7,05
Total	156	100,00
Você tem algum problema de saúde?		
Não	59	39,07
Sim	92	60,93
Total	151	100,00
Sintomas depressivos		
Não	20	12,66
Sim	138	87,34
Total	158	100,00
Sintomas de ansiedade		
Mínima	40	25,32
Leve	30	18,99
Moderada	34	21,52
Grave	54	34,17
Total	158	100,00
Transtornos mentais comuns		
Negativo	36	22,78
Positivo	122	77,22
Total	158	100,00

A avaliação dos instrumentos utilizados neste estudo permitiu identificar que a maioria dos participantes (60,93%) relatou algum problema de saúde. Entre os entrevistados, 87,34% apresentavam sintomas depressivos, 55,7% tinham ansiedade de moderada a grave e 77,22% foram positivos para TMC (Tabela 1).

De acordo com as perguntas realizadas para analisar a percepção atual de mudanças que aconteceram na vida das pessoas antes e após a realocação, foi possível identificar que houve uma piora percebida estatisticamente significativa na renda, no número de pessoas que moram na sua casa, na sua saúde física e mental, no acompanhamento psicológico e psiquiátrico, na ideação suicida e na forma como via sua vida (Tabela 2). É possível verificar que, após o evento de realocação dos moradores, a renda relatada diminuiu em todas as faixas identificadas no estudo. Na percepção dos entrevistados, sua saúde física e mental era considerada boa e muito boa e, após a realocação, passou a ser média, ruim e muito ruim. Houve um aumento de 35,94% dos casos de pessoas que passaram a fazer acompanhamento psicológico e de 26,9% dos que passaram a fazer acompanhamento psiquiátrico após a realocação, segundo a percepção dos participantes do estudo. Ainda mais preocupante é o aumento percebido de 25,65% de pessoas com ideação suicida. Identificou-se que a forma como os entrevistados veem sua vida foi comprometida, quando se compara sua percepção antes e após a realocação, tendo em vista que consideravam sua vida muito boa e boa e hoje a consideram média, ruim e muito ruim (Tabela 2).

**Tabela 2:** Percepção das mudanças que aconteceram na vida das pessoas antes e após serem vítimas da instabilidade do solo nos bairros afetados pela extração de sal-gema de uma mineradora de Maceió, Alagoas, Brasil, 2023.

Variáveis	Antes		Após		Valor de p
	n	%	n	%	
Renda mensal familiar (salário mínimo) *					< 0,01
Menos de 1	10	6,76	24	16,22	
De 1 até 3	45	30,41	48	32,43	
Mais de 3 até 5	42	28,38	35	23,65	
Mais de 5	51	34,46	41	27,70	
Número de pessoas que moravam/moram em sua casa					< 0,01
1	7	4,52	10	6,45	
2	24	15,48	38	24,52	
3	47	30,32	54	34,84	
4	41	26,45	33	21,29	
5 ou mais	36	23,23	20	12,90	
Como você considerava/considera a sua saúde física					< 0,01
Muito ruim	1	0,64	29	18,59	
Ruim	5	3,21	49	31,41	
Média	20	12,82	55	35,26	
Boa	63	40,38	20	12,82	
Muito boa	67	42,95	3	1,92	
Como você considerava/considera a sua saúde mental					< 0,01
Muito ruim	3	1,91	40	25,48	
Ruim	3	1,91	59	37,58	
Média	13	8,28	45	28,66	
Boa	63	40,13	11	7,01	
Muito boa	75	47,77	2	1,27	
Você fazia/faz algum acompanhamento psicológico					< 0,01
Sim	27	17,65	82	53,59	
Não	126	82,35	71	46,41	
Você fazia/faz algum acompanhamento psiquiátrico					< 0,01
Sim	12	7,89	53	34,87	
Não	140	92,11	99	65,13	
Você tinha/tem pensamentos de tirar sua vida					< 0,01
Sim	7	4,61	46	30,26	
Não	145	95,39	106	69,74	
Como você considerava/considera a sua vida					< 0,01
Muito ruim	2	1,27	20	12,74	
Ruim	0	0,00	33	21,02	
Média	5	3,18	70	44,59	
Boa	48	30,57	30	19,11	
Muito boa	102	64,97	4	2,55	

Nota: teste de McNemar.

* Foi considerado o salário mínimo de R$ 1.100,00.

Constatou-se que, mediante a percepção dos participantes, aqueles com maior demanda de acompanhamento psicológico (RP = 1,16; IC95%: 1,04-1,3; p < 0,001) e psiquiátrico (RP = 1,15; IC95%: 1,04-1,28; p < 0,001), ideação suicida (RP = 1,17; IC95%: 1,07-1,29; p < 0,001) e que consideravam que sua vida tinha piorado (RP = 1,64; IC95%: 1,08-2,49; p < 0,001) após a realocação de suas residências apresentaram prevalência mais elevada de sintomas depressivos (Tabela 3).

**Tabela 3:** Razão de prevalência (RP) da ocorrência de sintomas depressivos nas pessoas vítimas da instabilidade do solo nos bairros afetados pela extração de sal-gema de uma mineradora de Maceió, Alagoas, Brasil, 2023.

Mudanças	Sintomas depressivos					
	Não tem risco		Tem risco		RP	IC95%
	n	%	n	%		
Renda						
Não piorou	14	14,43	83	85,57	Referência	
Piorou	5	9,80	46	90,20	1,05	0,93-1,19
Número de moradores						
Não piorou	14	10,00	126	90,00	Referência	
Piorou	5	33,33	10	66,67	0,74	0,52-1,06
Saúde física						
Não piorou	7	35,00	13	65,00	Referência	
Piorou	13	9,56	123	90,44	1,39	1,00-1,93
Saúde mental						
Não piorou	3	30,00	7	70,00	Referência	
Piorou	17	11,56	130	88,44	1,26	0,84-1,90
Acompanhamento psicológico						
Não piorou	16	17,98	73	82,02	Referência	
Piorou	3	4,69	61	95,31	1,16	1,04-1,30
Acompanhamento psiquiátrico						
Não piorou	18	16,98	88	83,02	Referência	
Piorou	2	4,35	44	95,65	1,15	1,04-1,28
Ideação suicida						
Não piorou	19	16,96	93	83,04	Referência	
Piorou	1	2,50	39	97,50	1,17	1,07-1,29
Sua vida						
Não piorou	8	44,44	10	55,56	Referência	
Piorou	12	8,63	127	91,37	1,64	1,08-2,49

IC95%: intervalo de 95% de confiança.

Nota: modelo de regressão de Poisson com variância robusta.

Mediante a percepção dos participantes, aqueles com maior demanda de acompanhamento psicológico (RP = 1,75; IC95%: 1,13-2,73; p < 0,001) e psiquiátrico (RP = 1,83; IC95%: 1,20-2,79; p < 0,001) e ideação suicida (RP = 1,96; IC95%: 1,28-3,00; p < 0,001) após a realocação de suas residências apresentaram prevalência mais elevada de sintomas de ansiedade grave (Tabela 4).

**Tabela 4:** Razão de prevalência (RP) da ocorrência de ansiedade das pessoas vítimas da instabilidade do solo nos bairros afetados pela extração de sal-gema de uma mineradora de Maceió, Alagoas, Brasil, 2023.

Mudanças	Sintomas de ansiedade					
	Não grave		Grave		RP	IC95%
	n	%	n	%		
Renda						
Não piorou	67	69,07	30	30,93	Referência	
Piorou	32	62,75	19	37,25	1,20	0,76-1,92
Número de moradores						
Não piorou	91	65,00	49	35,00	Referência	
Piorou	11	73,33	4	26,67	0,76	0,32-1,82
Saúde física						
Não piorou	16	80,00	4	20,00	Referência	
Piorou	87	63,97	49	36,03	1,80	0,73-4,45
Saúde mental						
Não piorou	6	60,00	4	40,00	Referência	
Piorou	98	66,67	49	33,33	0,83	0,38-1,84
Acompanhamento psicológico						
Não piorou	66	74,16	23	25,84	Referência	
Piorou	35	54,69	29	45,31	1,75	1,13-2,73
Acompanhamento psiquiátrico						
Não piorou	77	72,64	29	27,36	Referência	
Piorou	23	50,00	23	50,00	1,83	1,20-2,79
Ideação suicida						
Não piorou	82	73,21	30	26,79	Referência	
Piorou	19	47,50	21	52,50	1,96	1,28-3,00
Sua vida						
Não piorou	15	83,33	3	16,67	Referência	
Piorou	88	63,31	51	36,69	2,20	0,77-6,33

IC95%: intervalo de 95% de confiança.

Nota: modelo de regressão de Poisson com variância robusta.

Mediante a percepção dos participantes, aqueles que relataram piora na sua saúde física (RP = 1,81; IC95%: 1,11-2,96; p < 0,001), maior demanda de acompanhamento psiquiátrico (RP = 1,24; IC95%: 1,06-1,45; p < 0,001), ideação suicida (RP = 1,42; IC95%: 1,24-1,62; p < 0,001) e que consideravam que sua vida tinha piorado após a realocação de suas residências (RP = 1,61; IC95%: 1,01-2,58; p < 0,001) apresentaram prevalência mais elevada de serem positivos para TMC (Tabela 5).

**Tabela 5:** Razão de prevalência (RP) da ocorrência de transtornos mentais comuns (TMC) das pessoas vítimas da instabilidade do solo nos bairros afetados pela extração de sal-gema de uma mineradora de Maceió, Alagoas, Brasil, 2023.

Mudanças	TMC					
	Negativo		Positivo		RP	IC95%
	n	%	n	%		
Renda						
Não piorou	21	21,65	76	78,35	Referência	
Piorou	13	25,49	38	74,51	0,95	0,79-1,15
Número de moradores						
Não piorou	27	19,29	113	80,71	Referência	
Piorou	7	46,67	8	53,33	0,66	0,41-1,07
Saúde física						
Não piorou	11	55	9	45,00	Referência	
Piorou	25	18,38	111	81,62	1,81	1,11-2,96
Saúde mental						
Não piorou	4	40,00	6	60,00	Referência	
Piorou	32	21,77	115	78,23	1,30	0,78-2,18
Acompanhamento psicológico						
Não piorou	24	26,97	65	73,03	Referência	
Piorou	11	17,19	53	82,81	1,13	0,96-1,34
Acompanhamento psiquiátrico						
Não piorou	30	28,30	76	71,70	Referência	
Piorou	5	10,87	41	89,13	1,24	1,06-1,45
Ideação suicida						
Não piorou	35	31,25	77	68,75	Referência	
Piorou	1	2,50	39	97,50	1,42	1,24-1,62
Sua vida						
Não piorou	9	50,00	9	50,00	Referência	
Piorou	27	19,42	112	80,58	1,61	1,01-2,58

IC95%: intervalo de 95% de confiança.

Nota: modelo de regressão de Poisson com variância robusta.

## Discussão

Este estudo revelou elevada frequência de ideação suicida, sintomas depressivos, ansiedade moderada e grave, além de rastreio positivo para TMC na amostra investigada. Após serem realocados devido à instabilidade do solo, houve piora percebida, estatisticamente significativa, na renda mensal, saúde física, mental e na forma como veem sua vida. Além disso, são pessoas que alegam gastar mais tempo para ir de sua atual residência ao seu trabalho e maior demanda de acompanhamento psicológico e psiquiátrico. Estudo de Fernandes et al. ^11^, realizado com equipes de saúde da família no Vale do Itajaí, Santa Catarina, revelou que demandas de suporte psicológico foram as necessidades predominantes de cuidados no período pós-desastre.

Outros estudos destacam a relação entre os desastres e os impactos na saúde mental dos afetados, evidenciando sintomas de estresse pós-traumático, depressão, ansiedade e ideação suicida ^27^ ^,^ ^28^ ^,^ ^29^ ^,^ ^30^ ^,^ ^31^.

O estudo *PRISMMA: Pesquisa sobre a Saúde Mental das Família Atingidas pelo Rompimento da Barragem de Fundão em Mariana* ^27^ investigou os impactos do rompimento da barragem do Fundão, em Mariana, na saúde mental das famílias afetadas pelo desastre. Foram identificados impactos psicológicos (sintomas de transtorno de estresse pós-traumático, ansiedade, depressão e outras formas de sofrimento psicológico); problemas de saúde física exacerbados pelas condições de vida precárias após o desastre; perda de recursos (a perda de propriedades, meios de subsistência e laços comunitários contribuiu significativamente para o sofrimento das famílias); relações familiares e comunitárias (importância das redes de apoio social e familiar na mitigação dos impactos negativos e tensões e conflitos emergentes devido ao estresse prolongado).

Estudo que investigou a prevalência de sintomas psiquiátricos e os fatores associados na população adulta afetada pelo rompimento da barragem de rejeitos em Mariana, identificou alta prevalência de sintomas de depressão, ansiedade e estresse pós-traumático. A falta de suporte social adequado foi um fator importante na exacerbação dos sintomas. Além disso, pessoas com histórico prévio de problemas de saúde mental apresentaram maior vulnerabilidade para desenvolver novos sintomas após o desastre ^28^.

É preciso destacar que as pessoas que relataram uma percepção de maior demanda de acompanhamento psicológico e psiquiátrico, ideação suicida e que consideravam que sua vida tinha piorado após a realocação de suas residências apresentaram prevalência mais elevada de sintomas depressivos. Pessoas com percepção de maior demanda de acompanhamento psicológico, psiquiátrico e ideação suicida apresentaram prevalência mais elevada de sintomas de ansiedade grave. Aquelas com percepção de piora na sua saúde física, maior demanda de acompanhamento psiquiátrico, ideação suicida e que consideravam que sua vida tinha piorado após a realocação de suas residências apresentaram prevalência mais elevada de ser positivo para TMC.

Estudo de revisão analisou resultados de pesquisas que totalizaram aproximadamente 60 mil vítimas de desastres, durante o período de 20 anos (1981 a 2001). Os desfechos analisados incluíram uma variedade de impactos na saúde mental, tais como problemas psicológicos (p.ex.: transtorno de estresse pós-traumático, depressão e ansiedade), problemas de saúde em geral, problemas crônicos na vida cotidiana, perda de recursos e problemas específicos nos jovens. Estressores secundários, problemas psiquiátricos prévios e recursos psicossociais fracos ou em deterioração foram associados a maiores impactos negativos na vida ^29^.

Pesquisa realizada com a população de uma área afetada pelo rompimento da barragem de rejeitos em Brumadinho, identificou alta prevalência de uso de medicamentos psicotrópicos. Os autores apontam que o uso de psicotrópicos pode refletir no impacto psicossocial do desastre na comunidade, incluindo sintomas de ansiedade, depressão e estresse pós-traumático e destacam a necessidade de intervenções de saúde mental direcionada a essa população ^31^.

No desastre ocorrido em Maceió, as pessoas perderam uma cadeia econômica formada por pequenas e médias empresas que existiam nesses bairros, perderam sua vizinhança, seu território de identidade e sofreram impactos em suas condições de vida, saúde física e mental. Estudo realizado com a população vítima do rompimento da barragem de Brumadinho mostra que alguns discursos narrativos estão vinculados ao sofrimento acarretado pela alteração da estrutura socioafetiva e geográfica da cidade. Eles apontam que a cidade não é mais a mesma, na medida em que o local onde foram edificadas histórias, sonhos e planos já não existia mais, demandando a necessidade de elaborar o luto pela cidade perdida ^32^, fato semelhante ao que aconteceu no desastre de Maceió.

Vários aspectos inerentes aos desastres socioambientais podem ter contribuído para a elevada prevalência de adoecimento mental identificada. Estudo realizado com a população de Brumadinho mostra que desastres são eventos que resultam em séria interrupção do funcionamento de uma comunidade ou sociedade, afetando seu cotidiano. Envolve perdas materiais e econômicas e danos ambientais e à saúde das populações ^33^. Em Maceió, houve danos relacionados à redução da mobilidade da população; perda de equipamentos de saúde e educação; perda de memória e abandono ou demolição de pontos históricos da cidade; desvalorização de imóveis próximos às áreas atingidas e ao déficit habitacional que inflacionou o mercado imobiliário ^12^.

O desastre de Maceió ainda está em curso, e os impactos sociais e econômicos mais evidentes foram a desativação de todos os serviços públicos, empresas e residências nos bairros afetados, gerando desocupação e realocação para outras localidades ^34^. Os impactos e danos dos desastres ambientais vão além dos econômicos, com impactos sobre a infraestrutura e os recursos que servem de suporte aos serviços, comprometendo a capacidade de oferta de serviços de saúde para a população afetada ^35^.

Esse desastre causado pela instabilidade das minas de sal-gema em Maceió configura-se como um dos maiores desastres socioambientais em áreas urbanas, atualmente no mundo ^34^. Ele não ocasionou diretamente a morte de pessoas, mas gerou sofrimento às vítimas mediante a necessidade emergencial de realocação de suas residências devido ao processo de afundamento. A ausência de fatalidade parece ter atenuado a sensibilidade das pessoas em relação aos impactos desse desastre a essa população. A preocupação predominante está centrada nas compensações financeiras para suas habitações. Todavia, é fundamental reconhecer que as implicações da realocação vão muito além dos aspectos monetários, os efeitos desse desastre incidem sobre a história de vida das vítimas.

Em 25 de janeiro de 2019, uma barragem da empresa Vale S.A., em Brumadinho, rompeu-se, atingindo a área administrativa da empresa e a área rural em seu entorno. Esse rompimento desencadeou a morte direta de muitas pessoas e deixou outras desabrigadas. Muitos foram os desafios para que o Sistema Único de Saúde (SUS) se adaptasse à nova conjuntura, caracterizada por sofrimento intenso e perdas socioafetivas, escassez de emprego e recursos financeiros, consequências ambientais e demanda por ressignificação de uma identidade da comunidade atingida. A articulação entre educação e assistência social mostrou-se importante e primordial ^32^.

Um estudo de revisão mostra as implicações para a prática clínica e para a formulação de políticas públicas voltadas às vítimas de desastres. Na prática clínica, tem-se: a necessidade de desenvolver intervenções de saúde mental que levem em conta os fatores de risco específicos e a vulnerabilidade de diferentes grupos demográficos; e o fornecimento de apoio psicológico contínuo. Na formulação de políticas públicas, destaca-se que: a preparação e a resposta a desastres devem incorporar componentes da saúde mental; a alocação de recursos deve ser suficiente para fornecer suporte psicológico e social em longo prazo; e os profissionais de saúde e trabalhadores de resgate devem ser treinados para reconhecer e responder adequadamente aos problemas de saúde mental pós-desastre ^30^.

Outros estudos mostram a necessidade de se pensar em políticas de saúde mental que incluam suporte psicológico contínuo para as vítimas de desastres ^27^; programas de apoio psicológico contínuo, acessíveis e culturalmente apropriados para as comunidades afetadas; incorporação de componentes de saúde mental em planos de preparação e resposta a desastres para mitigar os impactos psicológicos futuros; capacitação de profissionais de saúde para reconhecer e tratar adequadamente os sintomas psiquiátricos relacionados a desastres; fortalecimento das redes de apoio social e comunitário ^28^ e sistemas de monitoramento contínuo da saúde mental nas áreas afetadas ^28^ ^,^ ^31^.

As ações de saúde pós-desastre, bem como os riscos à saúde mental das vítimas de desastres, em médio e longo prazos, geram uma série de desafios, tanto para os municípios como para o SUS ^33^. O papel dos profissionais de saúde mental, como enfermeiros psiquiátricos, assistentes sociais psiquiátricos e psicólogos clínicos, no cuidado às vítimas de desastres e o apoio institucional são muito importantes para sua recuperação ^36^.

Em Brumadinho, houve uma sensibilização das equipes de saúde do SUS sobre como elaborar estratégias psicossociais e de saúde mental para assistir a população. O município tinha 100% da população coberta pela Estratégia Saúde da Família e isso auxiliou na implantação da estratégia de atenção psicossocial e saúde mental. Isso permitiu a compreensão das rupturas sofridas pelas pessoas que estavam sendo cuidadas, e das ferramentas utilizadas para enfrentar situações de sofrimento, potencializando um cuidado ampliado e diminuindo o impacto na saúde mental ^37^.

Estratégias psicossociais desse âmbito não estão sendo realizadas no desastre de Maceió. Desde maio de 2021, existe um Centro de Acolhimento e Triagem (CAT) para oferecer serviços de assistência social e psicológica aos moradores dos bairros afetados pelo afundamento do solo em decorrência da atividade de exploração de sal-gema, realizada pela mineradora Braskem. Esse foi resultado do Termo de Cooperação Técnica elaborado pelos Ministérios Públicos Federal, Estadual e do Trabalho, no qual ficou acordado que a Braskem seria obrigada a custear a construção desse equipamento, que é administrado pelo município de Maceió ^38^.

O CAT está localizado em um dos bairros afetados pelo afundamento do solo e os ex-moradores estão distribuídos em diferentes bairros, aspecto que pode dificultar o acesso das vítimas aos serviços realizados nesse equipamento. O ideal seria que houvesse um acompanhamento descentralizado e preparado para receber essa população, sem que para isso fosse necessário ter que ir ao CAT para triagem e encaminhamento para a rede de atenção psicossocial do município, que já está saturada com as demandas da população em geral. É preciso se concentrar em estratégias de cuidado em saúde mental que permitam um acompanhamento psicossocial que seja de fácil acesso para esses ex-moradores.

Os resultados deste estudo devem ser interpretados considerando suas limitações: amostra intencional e não probabilística; não alcance de pessoas com nível de escolaridade mais baixo; dificuldade na adesão ao estudo devido ao analfabetismo e à falta de acesso à internet, ao celular e ao computador; considerar as percepções dos ex-moradores afetados pelo afundamento do solo. Como a amostra não foi escolhida aleatoriamente, os resultados não são representativos da população geral; pessoas com menor escolaridade podem ter perspectivas e experiências diferentes, que não foram identificadas no estudo, resultando em uma visão incompleta do problema; pessoas com menor acesso à tecnologia foram sub-representadas, comprometendo a equidade do estudo; as percepções dos ex-moradores podem ser altamente subjetivas e emocionalmente carregadas, o que pode ter influenciado nas respostas.

Apesar de suas limitações, o estudo é importante por se concentrar nas percepções dos ex-moradores afetados pelo afundamento do solo oferece *insights* detalhados sobre como esses indivíduos vivenciaram o problema, o que é crucial para entender o impacto do evento, uma vez que essas percepções fornecem uma compreensão mais holística do impacto do afundamento do solo. Dar voz a esses ex-moradores afetados permite que suas experiências sejam reconhecidas e consideradas nas soluções propostas, promovendo um senso de inclusão e participação nas decisões que os afetam diretamente.

Os achados têm potencial para contribuir na formulação de políticas e intervenções mais direcionadas e eficazes para os ex-moradores, para embasar estudos futuros que utilizem métodos mais robustos e amostras mais representativas, ajudando a identificar áreas que necessitam de investigação mais aprofundada e para aumentar a conscientização sobre os problemas enfrentados pelos ex-moradores, sensibilizando a sociedade e os formuladores de políticas para a necessidade de ações específicas e urgentes. É fundamental considerar com seriedade as suas necessidades de cuidados à saúde física e mental, principalmente ante os achados deste estudo, que revelam comprometimento da saúde associado à desocupação forçada de suas habitações.
